# Comparative Proteomics of Resistant and Susceptible Strains of *Frankliniella occidentalis* to Abamectin

**DOI:** 10.1002/elps.202400171

**Published:** 2025-01-09

**Authors:** Zahra Gholami, Foad Fatehi, Fatemeh Habibpour Mehraban, Paul A. Haynes, Khalil Talebi Jahromi, Vahid Hosseininaveh, Hadi Mosallanejad, Pär K. Ingvarsson, Naser Farrokhi

**Affiliations:** ^1^ Department of Cell & Molecular Biology, Faculty of Life Sciences and Biotechnology Shahid Beheshti University Tehran Iran; ^2^ Department of Plant Protection, College of Agriculture and Natural Resources University of Tehran Karaj Iran; ^3^ Department of Agriculture Payame Noor University (PNU) Tehran Iran; ^4^ Department of Molecular Sciences Macquarie University North Ryde Australia; ^5^ Iranian Research Institute of Plant Protection Agricultural Research Education and Extension Organization (AREEO) Tehran Iran; ^6^ Department of Plant Biology Swedish University of Agricultural Sciences Uppsala Sweden

**Keywords:** abamectin, differentially expressed protein, insecticide resistance, mechanism, metabolism, regulation

## Abstract

Western flower thrips, *Frankliniella occidentalis* (*Thysanoptera*: Thripidae) is an invasive agricultural pest with developed resistance to abamectin in some strains due to frequent treatment with the pesticide. In this study, we examined differentially expressed proteins (DEPs) between abamectin‐resistant (Aba^R^; under abamectin selective pressure) and susceptible strains (Aba^S^; without abamectin selective pressure) of *F. occidentalis*. Proteins were isolated from second instar larvae of both strains and separated via two‐dimensional polyacrylamide gel electrophoresis. Nano‐flow liquid chromatography–tandem mass spectrometry identified selected protein spot features. From 70 DEPs, 43 spot features were identified: A total of 23 showed an increase in abundance, and 20 were down‐regulated in response to abamectin pressure. The enzymatic and structural proteins were classified into the functional groups of macromolecular metabolisms, signaling and cellular processes, immune system, genetic information processing, and exoskeleton‐related proteins. The up‐regulation of exoskeleton‐related proteins may contribute to forming a thicker cuticle, potentially hindering abamectin penetration, which is an interesting finding that needs further investigation. Two novel proteins, triacylglycerol lipase and cuticle protein CPF 2, were only expressed in Aba^R^. This work provides insights into abamectin resistance mechanisms in *F. occidentalis*, which will provide important information for developing insecticide resistance management approaches for this pest.

## 1 Introduction

Western flower thrips (WFT), *Frankliniella occidentalis* (*Thysanoptera*: Thripidae), is an economically important insect pest due to its wide plant host range, high fecundity rate, short generation time, and overlap of generations that makes it difficult to control [1, 2]. *F. occidentalis* causes substantial economic loss by direct (feeding and laying) and indirect (transmission of the tospoviruses) damages [2]. WFT has a high potential to become resistant to insecticides due to polyphagy, thigmotactic behavior (preference to hide in parts of plants where pesticides may not reach and reduce the selective pressure of insecticides), and having a haplodiploid reproductive system [3]. WFT is, therefore, considered a hard‐to‐control pest, mainly due to the development of resistance to insecticides [4]. Different strains of WFT have shown resistance to various groups of insecticides [1, 5]. It has recently been documented that there are 176 cases of insecticide resistance in *F*. *occidentalis* strains from all over the world. More importantly, the reports showed that thrips larvae are less sensitive than adults to insecticides, and this resistance level remains stable in the larval stage [6]. Abamectin, a macrocyclic compound and a member of avermectins, can control mites, nematodes, insects, and greenhouse pests such as *F. occidentalis* [5]. Abamectin is a worldwide registered insecticide, and due to prolonged use, numerous pests have emerged resistant to it [7, 8, 9]. Therefore, the investigation of resistance mechanisms at the molecular level is vital to delay the development of abamectin resistance and to manage the control of the *F*. *occidentalis*–resistant population [10].

WFT has shown resistance to abamectin via different mechanisms, such as increased activity of cytochrome P450, mutations in glutamate and GABA‐gated chloride channels, down‐regulation of glutamate receptor genes, and decreased cuticular penetration [5, 7, 11, 12]. However, changes in other pathways that may affect the ability of the insect to overcome abamectin selective pressure have not yet been addressed. Proteomics can be the method of choice to pinpoint insecticide resistance mechanisms in pests, as it is considered the ultimate response [10]. Such findings have merits in developing transgenic resistant host plants benefiting from RNA interference (RNAi) technology or other similar means to silence the target genes involved in insecticide resistance in WFT [13]. In this study, we used a combination of two‐dimensional polyacrylamide gel electrophoresis (2‐DE) and nano‐flow liquid chromatography–tandem mass spectrometry (nLC–MS/MS) to isolate proteins involved in resistance to abamectin in WFT to understand the molecular mechanisms of resistance to this pesticide and to determine changes at the protein levels that were associated with the adaptation to abamectin. Accordingly, among 70 proteins with significant fold changes in an abamectin‐resistant strain (Aba^R^), 43 protein spots were successfully identified. The differentially expressed proteins (DEPs) were mainly categorized into carbohydrate, lipid, and energy metabolism; protein metabolism; immune system; and exoskeleton. The results are reviewed concerning known and novel resistance mechanisms conferring abamectin resistance in WFT, which will advance our understanding of the molecular basis of the mechanisms developed by this pest to resist abamectin selective pressure.

## 2 Materials and Methods

### 2.1 *F. occidentalis* Cultures

An abamectin‐resistant strain (Aba^R^) of *F*. *occidentalis* was collected from a strawberry greenhouse in Alborz, Iran. Aba^R^ was in a greenhouse in which abamectin was used more than 30 times in a single season, and the thrips were subjected to high selective abamectin pressures. Therefore, the probability of developing resistance to this insecticide was high. Thus, in each insecticide application, survived thrips supposedly developed resistance to abamectin and dominated the insect population. A reference susceptible strain (Aba^S^) of *F*. *occidentalis* was obtained from an isolated eggplant greenhouse without any selective pressure of insecticides from the Isfahan region, Iran. Thrips collected from both greenhouses were subsequently maintained on fresh green bean pods (*Phaseolus vulgaris*) at 25°C ± 2°C, 60% ± 5 relative humidity, and 16:8 h light:dark to be used in the experiments [4]. The bioassay method was used to estimate the resistance factor between Aba^R^ and Aba^S^ strains. For this purpose, bioassay experiments were performed using the method recommended by the Insecticide Resistance Action Committee (IRAC), number 014 (www.irac‐online.org), on second instar larvae of both strains. On the basis of the results, it was found that the resistance factor for Aba^R^ as compared to the Aba^S^ was about 60‐fold [14].

### 2.2 Chemicals

Trichloroacetic acid, acetone, dithiothreitol (DTT), urea, thiourea, CHAPS, ampholytes, Tris, sodium dodecyl sulfate (SDS), glycerol, Coomassie Brilliant Blue R250, and iodoacetamide were obtained from Sigma Chemical Company (St. Louis, Missouri, USA). IPG (immobilized pH gradient) gel strips were purchased from Bio‐Rad (California, USA). Acetonitrile (ACN), NH_4_HCO_3_, trypsin, and formic acid were from Merck (Kenilworth, New Jersey, USA).

### 2.3 Protein Isolation

The synchronized second instar larvae from the fourth generation of both Aba^R^ and Aba^S^ strains were used for protein isolation. In total, 3000 insects for each strain, in 3 replicates, were powdered in liquid nitrogen, and protein was isolated by precipitation at 4°C by 10% (w/v) trichloroacetic acid/acetone containing 0.07% (w/v) DTT. The tubes were vigorously vortexed, kept at −20°C for 16 h, centrifuged at 18 000 × *g* at 4°C for 20 min, and the resulting pellet was washed four times with cold acetone containing 0.07% (w/v) DTT. The pellet was air‐dried and solubilized in 400 µL rehydration sample buffer (8 M urea, 2 M thiourea, 4% (w/v) CHAPS, and 0.2% (w/v) ampholyte 3–10). The pellet was vortexed at 22°C for 1 h and centrifuged at 18 000 × *g* (15°C) for 30 min. The supernatant was collected and kept at −80°C until electrophoresis.

### 2.4 Two‐Dimensional Gel Electrophoresis (2DE)

IPG gel strips with 18 cm length and pH range between 3 and 10 were rehydrated with rehydration buffer (8 M urea, 2 M thiourea, 50 mM DTT, 4% (w/v) CHAPS, and 0.2% (w/v) ampholytes 3–10) for 16 h. Isoelectric focusing (IEF) was performed in Amersham GE Healthcare Ettan IPGphor using a voltage step‐gradient program [250 V: 2000 Vh; 8000 V: 18 000 Vh; 8000 V: 20 000 Vh] at 20°C, and a maximum current setting of 70 A per strip was applied. The IPG strips were equilibrated for 15 min in equilibration buffer I (0.5 M Tris buffer containing 6 M urea, 4% (w/v) SDS, 30% (v/v) glycerol, 1% (w/v) DTT, pH 8.8), followed by 15 min in equilibration buffer II (0.5 M Tris buffer containing 6 M urea, 4% (w/v) SDS, 30% (v/v) glycerol, 5% (w/v) iodoacetamide, pH 8.8). The second‐dimension electrophoresis was carried out on a 12.5% polyacrylamide gel at a constant voltage of 75 V. The protein spots were stained using Coomassie Brilliant Blue R250 and scanned using an Image Scanner III (GE Healthcare) and analyzed using Image Master 2D Platinum 7.0 (GE Healthcare). Spots were detected by Melanie software 6.02 (GeneBio, Geneva, Switzerland) for gels of both the Aba^R^ and Aba^S^ strains. Analysis of variance (ANOVA) was performed, and the means were compared (*p* ≤ 0.05). Spots that showed a change of ≥0.49‐fold in average spot volume out of 3 replicates (a total of 46 spots) between Aba^R^ and Aba^S^ strains were excised and used for sequencing via mass spectrometer.

### 2.5 Protein Identification by MS/MS

DEP spots were washed with 100 mM NH_4_HCO_3_ to adjust pH and washed with 200 µL (1:1) acetonitrile (ACN):50 mM NH_4_HCO_3_ until the blue color faded [15]. The wash was repeated once more for 5 min, followed by 5 min wash with ACN. The samples were air‐dried for 10 min and reduced with 50 µL of 10 mM DTT in 100 mM NH_4_HCO_3_ for 60 min at 37°C. The gel pieces were alkylated with 50 µL of 55 mM iodoacetamide in 100 mM NH_4_HCO_3_ for 45 min in dark at 22°C. Subsequently, the gel pieces were washed with 100 µL of 100 mM NH_4_HCO_3_ and twice with 1:1 ACN:50 mM NH_4_HCO_3_ for 5 min, dehydrated with 100 µL of 50% can, and air‐dried. Proteins were digested with 20 of 12.5 ng/µL trypsin in 50 mM NH_4_HCO_3_ at 37°C for 16 h. Proteins were extracted in 30 µL of 50% ACN/2% formic acid and dried in a vacuum centrifuge. Peptides were reconstituted in 10 µL of 1% (v/v) formic acid.

### 2.6 Nano‐Flow Liquid Chromatography–Tandem Mass Spectrometry

Protein spots were analyzed by nLC‐MS/MS using a Q Exactive Orbitrap mass spectrometer coupled to an EASY‐nLC1000 nanoflow HPLC system (Thermo Scientific, San Jose, CA, USA). The spots were separated on a C18 column and injected into a mass spectrometer [15]. Reversed‐phase chromatographic separation was carried out on a column with a 75 µm internal diameter packed in‐house to 10 cm length with ES‐C18 Halo, 2.7 µm bead size, 160 Å pore size (Advanced Materials Technology, Wilmington, DE, USA) in a fused silica capillary using an integrated electrospray tip. All triplicates of excised spots were measured, each in a 1 h nLC gradient. A 1 h linear solvent gradient, starting with 100% Buffer A (0.1% formic acid), with steps from 0% to 40% of Buffer B (99.9% (v/v) ACN, 0.1% (v/v) formic acid) over 50 min and 40% to 85% of Buffer B over 10 min, was applied to elute peptides from the C18 column. MS/MS was performed in the data‐dependent acquisition (DDA) mode with MS/MS of the top 10 most abundant precursor ions at higher energy C‐trap dissociation (HCD) normalized collision energy of 35%. Xcalibur software (version 2.06) (Thermo, Fremont, CA, USA) was used to perform spectral acquisition over the mass range of 400–1500 *m*/*z*, automated peak recognition, detection of ions in the Orbitrap at a resolution of 70 000, HCD fragmentation of target ions, and dynamic exclusion of fragmented ions for 90 s.

### 2.7 Protein Identification

Raw data obtained from MS were converted to mzXML format and analyzed using the global proteome machine (GPM) software version 2.2.1 (https://www.thegpm.org/). MS/MS spectra were used for peptide‐to‐spectrum matching against an *F. occidentalis* protein database, downloaded in September 2020 from the Universal Protein Resource (http://www.uniprot.org/; Uniprot) [16]. The search parameters included 0.4 Da fragment mass tolerance and K/R‐P cleavages, cysteine carbamidomethylation for fixed and methionine oxidation for variable modifications. Proteins with log (e)^+^ values of <−30 and at least seven spectral counts were retained for further analysis.

### 2.8 Bioinformatics Analysis

Orthologous sequences of DEP spots for WFT were found in *Drosophila melanogaster* and used for functional annotation in Uniprot, KEGG (https://www.genome.jp/kegg/) [17], and STRING (http://www.string‐db.org/) [18]. Subcellular localizations of proteins were analyzed by BUSCA (http://busca.biocomp.unibo.it/) [19]. AgriGO (http://bioinfo.cau.edu.cn/agriGO/analysis.php) was used for gene ontology (GO) [20].

## 3 Results

### 3.1 Comparative Proteomics of Aba^R^ and Aba^S^ Strains in Response to Abamectin

**FIGURE 1:**
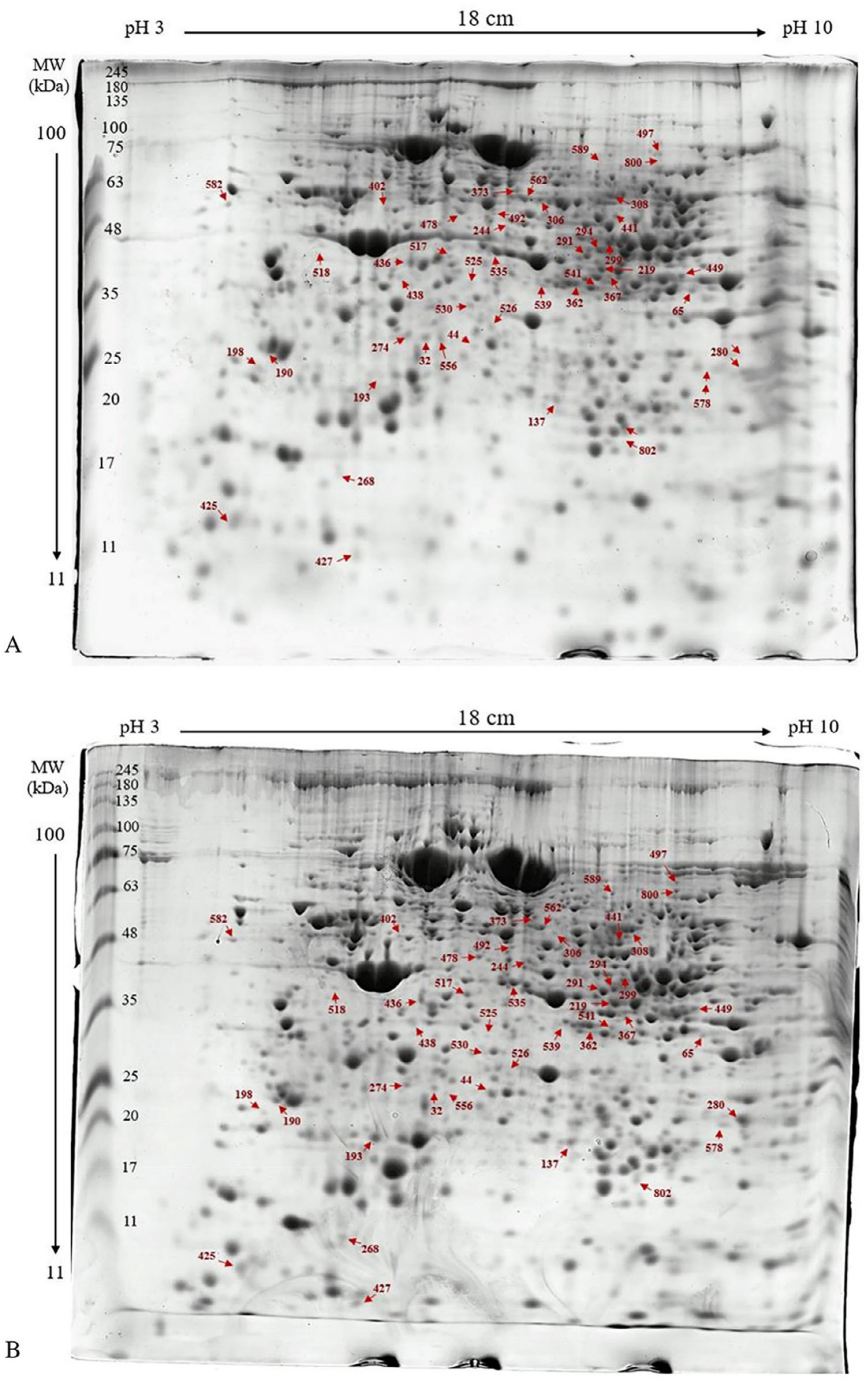
Two‐dimensional gel electrophoresis of proteins of *Frankliniella occidentalis* second instar larvae: (A) representative gel of susceptible strain (Aba^S^) and (B) representative gel of resistant strain (Aba^R^). Total protein (400 µg) extracted from a pool of 1000 2nd instar larvae of *F. occidentalis* on immobilized pH 3–10 non‐linear IPG strips in the first dimension with 3 protein extracts for each strain. The second dimension was on 12.5% SDS–PAGE gels. A number of DEP spots were 70, and 43 were identified by nLC–MS/MS. Among all identified protein spots, 23 showed increased in abundance and 20 showed decreased in abundance. Red spots represent DEPs between Aba^R^ and Aba^S^ strains. Molecular mass (in kilo Daltons) is shown on the *y* axis, and *p*I (as pH range) is shown on the *x* axis.

**FIGURE 2:**
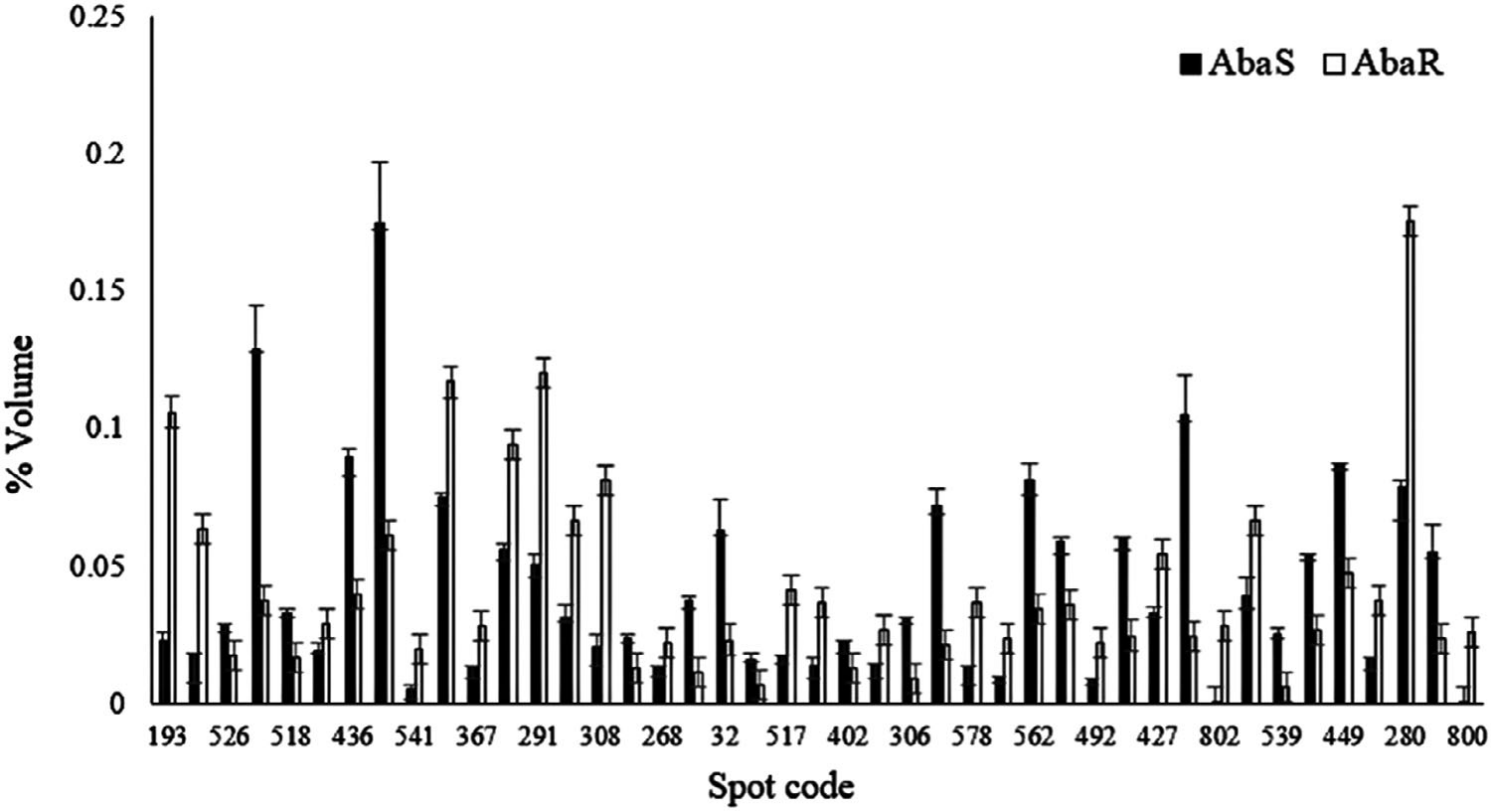
A representative histogram showing DEP spots in Aba^R^ and Aba^S^ strains of WFT.

Comparative proteomics was carried out between Aba^R^ and Aba^S^ strains of *F. occidentalis* to demonstrate differential protein expression patterns. Total protein (400 µg) was isolated from 1000 2nd instar larvae of WFT; a total of 585 protein spots were reproducibly visualized (Figure 1), and a total of 70 DEPs were found (*p* < 0.05) to have between 0.5‐ and 1.5‐fold change, 43 of which were excised for digestion and MS analyses (Figure 2, Table 1). Among these 43 spots, 23 and 20 showed increased or decreased abundance in Aba^R^ in comparison with those in Aba^S^ strain, respectively (Table 2). Four spots showed a presence/absence pattern in the Aba^R^ strain. Triacylglycerol lipase (Spot 802) and cuticle protein CPF 2 (800) were absent in Aba^S^ but expressed in Aba^R^, with 2.07‐ and 1.35‐fold changes, respectively. Some spot IDs were reported twice, including pyruvate dehydrogenase, mitochondrial medium‐chain‐specific acyl‐CoA dehydrogenase, galactokinase, and phosphoenolpyruvate carboxykinase. The repetition might be due to the presence of different protein isoforms, post‐translational modification, or degradation (Table 2). Moreover, eight protein spots (526, 541, 497, 268, 198, 306, 578, and 478) were not identified, which may be due to insufficient protein being extracted or insufficient quantities of amenable peptides being produced. To identify the hypothetical and uncharacterized proteins, BLASTp was carried out to narrow down the probable function (Table 3). Proteins that increased or decreased in abundance in Aba^R^ compared to Aba^S^ are listed in Table 2 with the corresponding fold changes.

**TABLE 1:** Characteristics of differentially expressed protein (DEP) spots.

Protein name	Spot code	^a^Acc No.	^a^ANOVA	MW (kDa)	*^a^p*I/Mr^2^	^a^Log (e)
Cuticle protein CPR RR‐1 15	193	KAE8739716.1	0.0236	21.5	5.47/21.5	−56.4
Glycerol‐3‐phosphate dehydrogenase [NAD (+)], cytoplasmic isoform X1	44	XP_026293193.1	0.0116	38.9	5.86/38.9	−214.2
Hypothetical protein FOCC_FOCC002301	526	KAE8750873.1	0.0098	40.5	8.40/40.5	−71.5
Galectin‐4‐like	190	XP_026289802.1	0.0041	37.8	6.22/37.8	−291.3
Fructose‐1,6‐bisphosphatase isozyme 2	518	XP_026274903.1	0.0052	37.0	6.76/37	−208.7
Probable pyruvate dehydrogenase E1 component subunit alpha	582	XP_026283979.1	0.0082	43.2	8.48/43.2	−262.6
Medium‐chain‐specific acyl‐CoA dehydrogenase, mitochondrial	436	KAE8749078.1	0.003	46.1	8.36/46.1	−473.4
Medium‐chain‐specific acyl‐CoA dehydrogenase, mitochondrial	535	KAE8749078.1	0.0078	46.1	8.36/46.1	−157.5
Hypothetical protein FOCC_FOCC003800	541	KAE8749535.1	0.0254	28.6	7.06/28.6	−246.0
Adenylosuccinate lyase–like	362	XP_026275254.1	0.0002	54.5	6.44/54.5	−66.4
Cytosol aminopeptidase–like isoform X2	367	XP_026287108.1	0.0206	54.5	6.47/54.5	−177.7
Allergen Cr‐PI‐like	219	XP_026284806.1	0.001	84.9	5.78/84.9	−106.6
Probable pyruvate dehydrogenase E1 component subunit alpha, mitochondrial isoform X3	291	XP_026283979.1	0.0002	43.2	8.48/43.2	−394.9
FK506‐binding protein 2 isoform X2	441	XP_026273972.1	0.0022	24.1	4.83/24.1	−140.7
Cathepsin B–like peptidase 2	308	KAE8744205.1	0.0021	36.0	6.70/36.0	−141.6
Hypothetical protein FOCC_FOCC008316	497	KAE8745065.1	0.0052	46.1	5.49/46.1	−158.9
Uncharacterized protein LOC113211199	268	XP_026285291.1	0.0386	34.5	5.64/34.5	−456.2
Hypothetical protein FOCC_FOCC012065	198	KAE8742378.1	0.0178	31.3	6.98/31.3	−76.8
3(2),5‐bisphosphate nucleotidase 1	32	XP_026280724.1	0.0303	33.2	5.65/33.2	−161.7
Ecdysteroid kinase 4	525	KAE8739877.1	0.0404	48.4	5.31/48.4	−76.8
Aminoacylase‐1‐like isoform X1	517	XP_026290178.1	0.0017	45.1	5.93/45.1	−101.1
Galactokinase‐like	438	XP_026286793.1	0.0118	46.0	8.14/46	−532.4
Proliferation‐associated protein 2G4	402	XP_026277136.1	0.003	42.9	6.62/42.9	−110.0
Galactokinase‐like	244	XP_026286793.1	0.0512	46.0	8.14/46.0	−532.4
Uncharacterized protein LOC113211558	306	XP_026285747.1	0.0002	27.0	8.60/27.0	−65.5
ATP‐dependent RNA helicase	299	KAE8741845.1	0.0017	79.0	6.48/79.0	−27.7
Hypothetical protein FOCC_FOCC017905	578	KAE8736640.1	0.0308	86.1	5.72/86.1	−149.7
Putative aldehyde dehydrogenase family 7 member A1 homolog	589	XP_026275375.1	0.0025	57.7	7.42/57.7	−222.6
14‐3‐3‐ zeta	562	KAE8742788.1	0.004	24.5	9.13/24.5	−178.2
Hypothetical protein FOCC_FOCC000031	478	KAE8753108.1	0.0058	37.8	7.55/37.8	−123.3
Aldose reductase–like	492	XP_026286026.1	0.0023	36.2	5.92/36.2	−164.2
Galectin‐9‐like isoform X2	373	XP_026275085.1	0.0007	36.8	8.20/36.8	−230.9
Fructose‐bisphosphate aldolase‐like	427	XP_026277347.1	0.0011	36.5	8.40/36.5	−247.7
Alpha‐glucosidase	425	KAE8736515.1	0.0057	52.0	5.36/52.0	−22.4
Pancreatic triacylglycerol lipase	802	KAE8750744.1	0.0431	39.2	5.48/39.2	−11.8
Larval/Pupal cuticle protein H1C	137	XP_026272997.1	0.0242	23.4	6.91/23.4	−311.3
Brachyurin‐like	539	XP_026294113.1	0.0002	38.6	8.07/38.6	−100.6
Phosphoenolpyruvate carboxykinase	65	KAE8751936.1	0.0018	72.2	7.88/72.7	−157.0
Phosphoenolpyruvate carboxykinase	449	KAE8751936.1	0.0088	72.7	7.88/72.7	−157.0
UV excision repair protein RAD23 homolog B isoform X5	414	XP_026284341.1	0.0045	32.7	4.40/32.7	−104.9
Alcohol dehydrogenase	280	XP_026275521.1	0.0012	36.6	5.36/36.6	−279.9
L‐Lactate dehydrogenase	274	XP_026292259.1	0.0359	37.6	7.26/37.6	3.0
Cuticle protein CPF 2	800	KAE8752868.1	0.0108	63.2	6.90/63.2	4.0

*Note*: ANOVA values represent the mean/median of the triplicates. Spot codes, isoelectric point (*p*I), molecular weight, protein name, and identifiers are presented.

Abbreviation: ANOVA, analysis of variance.

^a^Acc No. = Accession number of proteins in NCBI. pI/Mr^2^: Isoelectric point/Relative mass, *ρ* = Log(*e*): The expectation values for the peptides are distributed as expected from random matching. ANOVA values from Image Master software.

**TABLE 2:** Identified protein spots in second instar larvae of an abamectin‐resistant strain (Aba^R^) strain, with fold expression values, functional categories/functional subcategories, and KEGG Brite.

Protein name	^a^Acc No.	^a^Fold change	Functional categories/functional subcategories (KEGG)	^a^KEGG brite
Cuticle protein CPR RR‐1 15	KAE8739716.1	+4.64	Exoskeleton	Structural constituent of cuticle
Glycerol‐3‐phosphate dehydrogenase	XP_026293193.1	+3.55	Lipid metabolism/glycerophospholipid metabolism	Enzyme/Oxidoreductases
Hypothetical protein FOCC_FOCC002301	KAE8750873.1	−0.63	—	—
Galectin‐4‐like	XP_026289802.1	−0.29	Signaling and cellular processes	Lectins *S*‐type lectins
Fructose‐1,6‐bisphosphatase isozyme 2	XP_026274903.1	−0.49	Carbohydrate metabolism/glycolysis—gluconeogenesis	Enzyme/Hydrolases
Probable pyruvate dehydrogenase E1 component subunit alpha	XP_026283979.1	+1.48	Carbohydrate metabolism/pyruvate metabolism	Enzyme/Oxidoreductases
Medium‐chain‐specific acyl‐CoA dehydrogenase, mitochondrial	KAE8749078.1	−0.44	Lipid metabolism/fatty acid degradation	Enzyme/Oxidoreductases
Medium‐chain‐specific acyl‐CoA dehydrogenase, mitochondrial	KAE8749078.1	−0.35	Lipid metabolism/fatty acid degradation	Enzyme/Oxidoreductases
Hypothetical protein FOCC_FOCC003800	KAE8749535.1	+3.64	—	—
Adenylosuccinate lyase–like	XP_026275254.1	+1.55	Nucleotide metabolism/purine metabolism	Enzyme/Lyases
Cytosol aminopeptidase–like isoform X2	XP_026287108.1	+2.17	Protein metabolism/arginine and proline metabolism—glutathione metabolism	Enzyme/Hydrolases
Allergen Cr‐PI‐like	XP_026284806.1	+1.67	Immune system	Nutrient reservoir activity
Probable pyruvate dehydrogenase E1 component subunit alpha, mitochondrial isoform X3	XP_026283979.1	+2.37	Carbohydrate metabolism/pyruvate metabolism	Enzyme/Oxidoreductases
FK506‐binding protein 2 isoform X2	XP_026273972.1	+2.12	Protein metabolism/chaperones and folding catalysts	Enzyme/Isomerases
Cathepsin B–like peptidase 2	KAE8744205.1	+4.01	Cellular processes/transport and catabolism	Cysteine‐type peptidase activity
Hypothetical protein FOCC_FOCC008316	KAE8745065.1	−0.55	—	—
Uncharacterized protein LOC113211199	XP_026285291.1	+1.67	—	—
Hypothetical protein FOCC_FOCC012065	KAE8742378.1	−0.3	—	—
3(2),5‐bisphosphate nucleotidase 1	XP_026280724.1	−0.37	Energy metabolism/sulfur metabolism	Metal ion binding
Ecdysteroid kinase 4	KAE8739877.1	−0.43	Insect growth/ecdysteroid metabolism	Enzyme/Kinase
Aminoacylase‐1‐like isoform X1	XP_026290178.1	+2.33	Protein metabolism/arginine biosynthesis	Enzyme/Hydrolases
Galactokinase‐like	XP_026286793.1	+2.64	Unclassified metabolism/*galactose* regulation	Enzyme/Transferases
Proliferation‐associated protein 2G4	XP_026277136.1	−0.57	Unclassified metabolism	DNA‐binding protein that is involved in growth regulation
Galactokinase‐like	XP_026286793.1	+1.91	Unclassified metabolism/*galactose* regulation	Enzyme/Transferases
Uncharacterized protein LOC113211558	XP_026285747.1	−0.29	—	—
ATP‐dependent RNA helicase	KAE8741845.1	−0.30	Nucleotide metabolism/secondary structures of RNA molecules	Enzyme/Hydrolases
Hypothetical protein FOCC_FOCC017905	KAE8736640.1	+2.71	—	—
Putative aldehyde dehydrogenase family 7 member A1 homolog	XP_026275375.1	+2.52	Carbohydrate metabolism/oxidation of aldehydes	Enzyme/Oxidoreductases
14‐3‐3‐ zeta	KAE8742788.1	−0.42	Signal transduction/binding proteins	Exosome
Hypothetical protein FOCC_FOCC000031	KAE8753108.1	−0.60	—	—
Aldose reductase–like	XP_026286026.1	+2.57	Unclassified metabolism/glucose metabolism	Enzyme/Oxidoreductases
Galectin‐9‐like isoform X2	XP_026275085.1	−0.41	Signaling and cellular processes/innate defense	Lectins/*S*‐type lectins
Fructose‐bisphosphate aldolase‐like	XP_026277347.1	+1.67	Carbohydrate metabolism/glycolysis—gluconeogenesis	Enzyme/Lyases
Alpha‐glucosidase	KAE8736515.1	−0.23	Carbohydrate metabolism/galactose metabolism	Enzyme/Hydrolases
Pancreatic triacylglycerol lipase	KAE8750744.1	+2.07	Lipid metabolism/glycerolipid metabolism	Enzyme/Hydrolases
Larval/Pupal cuticle protein H1C	XP_026272997.1	+1.72	Exoskeleton	Structural constituent of cuticle
Brachyurin‐like	XP_026294113.1	−0.24	Protein metabolism/collagen hydrolysis	Enzyme/Serine‐type endopeptidase
Phosphoenolpyruvate carboxykinase	KAE8751936.1	−0.49	Carbohydrate metabolism/glycolysis—gluconeogenesis	Enzyme/Lyases
Phosphoenolpyruvate carboxykinase	KAE8751936.1	−0.54	Carbohydrate metabolism/glycolysis—gluconeogenesis	Enzyme/Lyases
UV excision repair protein RAD23 homolog B isoform X5	XP_026284341.1	+2.37	Genetic information processing/folding, sorting, and degradation of protein‐nucleotide excision repair	Proteasome/Proteasome interacting proteins (PIPs)
Alcohol dehydrogenase	XP_026275521.1	+2.22	Carbohydrate metabolism/ethanol metabolism (oxidation of primary or secondary alcohols to aldehydes or ketones)	Enzyme/Oxidoreductases
L‐Lactate dehydrogenase	XP_026292259.1	−0.42	Carbohydrate metabolism/pyruvate metabolism	Enzyme/Oxidoreductases
Cuticle protein CPF 2	KAE8752868.1	+1.35	Exosceleton	Structural constituent of cuticle

*Note*: KEGG Brite is a collection of hierarchical classification systems capturing functional hierarchies of various biological objects (https://www.genome.jp/kegg/kegg3b.html). “+” is a proteins with increased in abundance in Aba^R^ strain compared to the Aba^S^ strain. “−” is a proteins with decreased in abundance in Aba^R^ strain compared to the Aba^S^ strain.

^a^Acc No. = Accession number of proteins in NCBI.

**TABLE 3:** Hypothetical protein spots in second instar larvae of an abamectin‐resistant strain (Aba^R^) strain and their alternatives with BLASTp.

Spot code	Protein name	Acc No.	Alternative (*Frankliniella occidentalis*)	Acc No.	Query cover (%)	Identity (%)
526	Hypothetical protein FOCC_FOCC002301	KAE8750873.1	methionine adenosyltransferase 2 subunit beta‐like	XP_026273942.1	85	100.00
497	Hypothetical protein FOCC_FOCC008316	KAE8745065.1	NADH dehydrogenase [ubiquinone] flavoprotein 2, mitochondrial	XP_026290797.1	55	99.57
541	Hypothetical protein FOCC_FOCC003800	KAE8749535.1	dihydropteridine reductase	XP_026285935.1	87	100.00
268	Uncharacterized protein LOC113211199	XP_026285291.1	CBB mannanase 1	KAE8741684.1	100	99.38
198	Hypothetical protein FOCC_FOCC012065	KAE8742378.1	ATP synthase subunit b, mitochondrial	XP_026281758.1	100	100.00
306	Uncharacterized protein LOC113211558	XP_026285747.1	dehydrogenase/Reductase SDR family member 4	XP_026276951.1	97	30.83
578	Hypothetical protein FOCC_FOCC017905	KAE8736640.1	ELKS/Rab6‐interacting/CAST family member 1‐like	XP_026281814.1	56	99.79
478	Hypothetical protein FOCC_FOCC000031	KAE8753108.1	Transaldolase	XP_026288389.1	97	100.00

### 3.2 Functional Annotation

**FIGURE 3:**
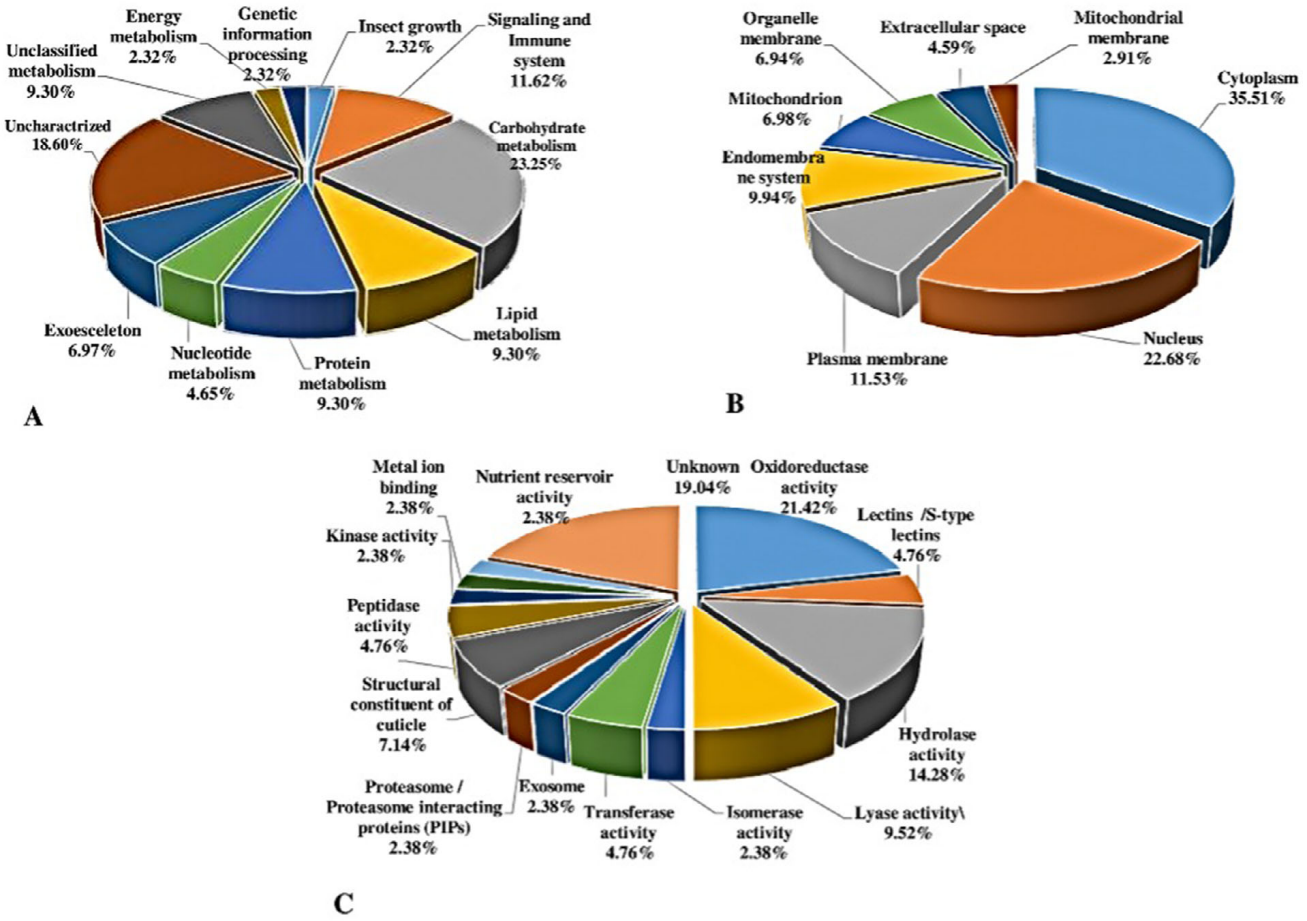
(A) Biological component of identified proteins from second instar larvae of WFT (Aba^R^ strain) by the Uniprot (http://www.uniprot.org/) and KEGG (https://www.genome.jp/kegg/); (B) cellular localization of identified proteins in Aba^R^ strain of WFT. Data were obtained from BUSCA as a subcellular localization predictor (http://busca.biocomp.unibo.it/) and AgriGO (http://bioinfo.cau.edu.cn/agriGO/analysis.php); (C) molecular function of identified proteins from Aba^R^ strain by the KEGG (https://www.genome.jp/kegg/) and Uniprot (http://www.uniprot.org/).

Proteins showing significant fold change in response to resistance to abamectin were grouped into 9 functional categories and 22 subcategories (Figure 3a–c, Table 2). The categories were cuticle metabolism (3 proteins), lipid metabolism (4 proteins), signaling, cellular processes, and defense response (5 proteins), carbohydrate metabolism (10 proteins), energy metabolism (1 protein), nucleotide metabolism and genetic information processing (3 proteins), protein metabolism (4 proteins), and insect growth (1 protein) (Figure 3a, Table 2). Molecular functions also were categorized into 14 groups, including oxidoreductase activity, lectins/*S*‐type lectins, hydrolase activity, lyase activity, isomerase activity, transferase activity, exosome, proteasome/proteasome‐interacting proteins (PIPs), structural constituents of the cuticle, peptidase activity, kinase activity, metal ion binding, nutrient reservoir activity, and unknown (Figure 3c).

### 3.3 Subcellular Localization

Subcellular localization of proteins was obtained in BUSCA (http://busca.biocomp.unibo.it/) and AgriGO (http://bioinfo.cau.edu.cn/agriGO/analysis.php). The results showed that 35.51% of DEPs are localized in the cytoplasm, 4.59% are being secreted to extracellular space, 22.68% are in the nucleus, 6.98% and 6.94% in mitochondrial and organellar membranes, 9.94% in the endomembrane system, and 11.53% in the plasma membrane (Figure 3b).

### 3.4 Protein–Protein Interaction

**FIGURE 4:**
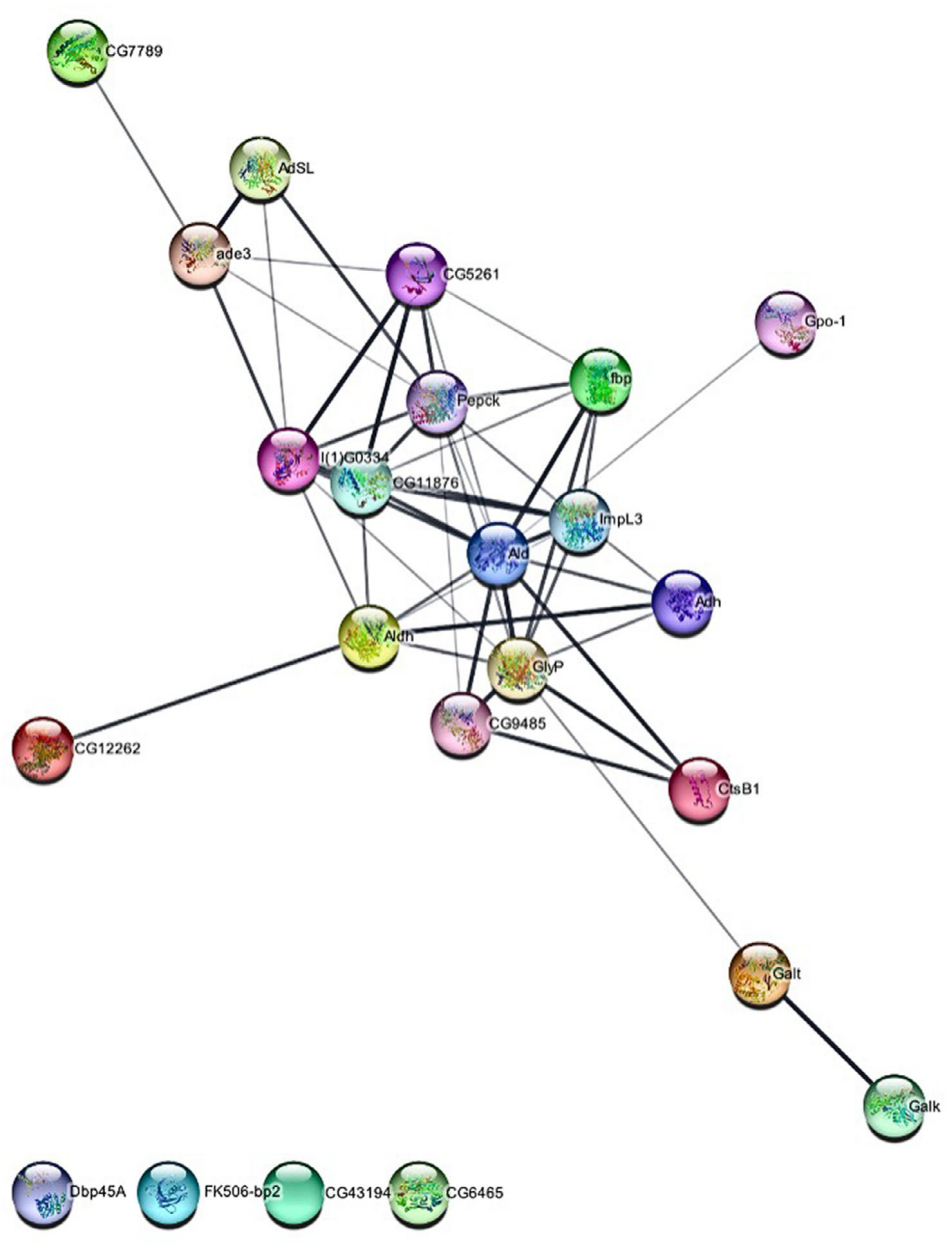
Protein–protein interaction (PPI). Identified proteins were used to survey the interaction between proteins. The results were obtained from the STRING (http://www.string‐db.org/). Interaction networks are shown in the confidence view in which stronger and weaker interactions are represented by thicker and dashed lines, respectively.

The protein–protein interaction (PPI) networks were generated in STRING for some of the proteins identified from the Aba^R^ strain (Figure 4). A network of high reliability (*p* value: 7.88e − 15) was obtained, with 23 nodes and 50 interactions. Two groups of functional interactions were noted. Members of the PPI networks belong to metabolic pathways, such as metabolisms of carbohydrates, lipids, and energy. These proteins are putative targets of abamectin pressure on the Aba^R^ strain and its resistance mechanisms against abamectin. Pyruvate dehydrogenase exhibited functional interactions with dihydrolipoyllysine‐residue acetyltransferase, L‐lactate dehydrogenase, aldehyde dehydrogenase, phosphoenolpyruvate carboxykinase, and fructose‐bisphosphate aldolase. Alcohol dehydrogenase showed functional interactions with aldehyde dehydrogenase, glycogen phosphorylase, fructose‐bisphosphate aldolase, and L‐lactate dehydrogenase. Galactokinase was shown to interact with probable galactose‐1‐phosphate uridylyltransferase. Phosphoenolpyruvate carboxykinase showed functional interactions with pyruvate dehydrogenase, L‐lactate dehydrogenase, fructose‐bisphosphate aldolase, fructose‐1,6‐bisphosphatase, and alpha‐1,6‐glucosidase. Aldehyde dehydrogenase exhibited functional interactions with probable medium‐chain‐specific acyl‐CoA dehydrogenase. Glycerol‐3‐phosphate dehydrogenase had interactions with fructose‐bisphosphate aldolase. However, four proteins, including aminoacylase, FK506‐binding protein (FKBP), 14‐3‐3, and ATP‐dependent RNA helicase, did not show any interactions with the groups described above, as each of these proteins was involved in a different biological pathway.

## 4 Discussion

To gain insights into the mechanisms involved in insecticide resistance, we analyzed changes in protein expressions in response to abamectin treatment in WFT. Differential expression of proteins may demonstrate the efficacy of insecticide pressure [21] and delineate interactions and coordination of different metabolic pathways in insects in response to insecticides [22]. A total of 27 DEPs were identified from 585 protein spots in a comparative study made between Aba^R^ and Aba^S^ strains of *F. occidentalis*. Of the 43 proteins identified, 23 and 20 proteins showed increased and decreased abundance in the resistant strain, respectively. On the basis of GO, KEGG database, and a literature survey, DEPs were divided into different subgroups correlating to their specific biological functions. The major protein subgroups in response to abamectin stress were proteins linked to carbohydrate and lipid metabolisms. Changes in the expression of any of the proteins in these catabolic cycles can affect energy production in the Aba^R^ strain, which could act as a fitness cost for insecticide resistance [23]. On the other hand, when DEPs were classified into eight categories according to subcellular localization, cytoplasmic proteins had the highest percentage (Figure 3b), and these proteins were mostly related to catabolic cycles. The proteins located in the extracellular space were linked to the cuticle and immune system. In contrast, the expression of immune system proteins in the nucleus decreased. Other responsive proteins in the nucleus include those involved in carbohydrate and nucleotide metabolisms. Mitochondrial‐responsive proteins were involved in carbohydrate, lipid, and protein metabolisms with probable roles in cellular respiration and energy production in the Aba^R^ strain.

### 4.1 Carbohydrate, Lipid, and Energy Metabolism Proteins

Fructose‐bisphosphate aldolase (Spot 427, EC 4.1.2.13) showed increased expression in Aba^R^, indicating a possible increase in the production of the corresponding metabolites, dihydroxyacetone phosphate and glyceraldehyde‐3‐phosphate, in the Calvin–Benson cycle, and this is something that warrants further investigation. Mating males of *Nilaparvata lugens* under triazophos treatment also showed a 2.11‐fold up‐regulation of this protein [24]. A strain of *Aphis gossypii*, known to be resistant to spirotetramat, showed roughly five‐fold higher expression of fructose‐bisphosphate aldolase compared to a susceptible strain [25]. In our study, Spot 478 (KAE8753108.1), with decreased abundance in the Aba^R^ strain, was identified as transaldolase with 97% query cover and 100% identity (XP_026288389.1). Due to the decreased abundance of Spot 478, this conversion is reduced, and glyceraldehyde‐3‐phosphate can be used in the Krebs cycle as a part of carbohydrate metabolism. Therefore, a relationship between an increased abundance of fructose‐1,6‐bisphosphate aldolase and a decreased abundance of transaldolase can be envisaged, leading to activation of the Calvin–Benson cycle. Zaluski et al. [26] also reported down‐regulation of transaldolase in nurse honeybees (*Apis mellifera*) heads exposed to pyraclostrobin and fipronil.

Pyruvate dehydrogenase (two isozymes: Spots 582 and 291, EC No. 2.3.1.12) showed an increased abundance in Aba^R^. Similarly, *A. mellifera* in response to nicotine [27] and a resistant strain of *Spodoptera frugiperda* in the presence of chlorpyrifos [28] demonstrated significant up‐regulation in both the corresponding enzyme and the gene, respectively.

Lactate dehydrogenase (Spot 274, EC 1.1.1.27) showed decreased abundance in the Aba^R^ strain compared to the control strain. Lactate dehydrogenase activity, an important glycolytic enzyme, has been reported to be hindered by insecticides [29].

Phosphoenolpyruvate carboxykinase (two isozymes: Spots 65 and 449, EC 4.1.1.32) was decreased in abundance in the Aba^R^ strain. Similarly, down‐regulation of the enzyme in response to cypermethrin was reported in Bacillus *thuringiensis* [30].

Glycerol‐3‐phosphate dehydrogenase [NAD^+^], cytoplasmic isoform X1 (Spot 44, EC 1.1.1.8), an antioxidant enzyme crucial in carbohydrate and lipid metabolism, showed increased abundance in the Aba^R^ strain. This enzyme is necessary for both carbohydrate and lipid metabolism by catalyzing the reversible reaction and production of FADH_2_ [31]. FADH_2_ is used in coenzyme Q in the mitochondria as an electron carrier to produce more ATP to overcome insecticide pressure and possibly to develop insecticide resistance [32]. In addition, the increased abundance of the enzyme in the Aba^R^ strain suggests the existence of a balance in NADH/NAD^+^ ratio. We thus hypothesized that the Aba^R^ strain, showing an increased abundance of glycerol‐3‐phosphate dehydrogenase, would have a more active antioxidant system compared to Aba^S^ strain. Similar to the Aba^S^ with a poor anti‐oxidative system, nurse bees exposed to fipronil and pyraclostrobin + fipronil demonstrated down‐regulation of this enzyme [26].

Pancreatic triacylglycerol lipase (Spot 802, EC 3.1.1.3), an enzyme related to lipid metabolism [33], was absent in the Aba^S^ strain. Lipases are most likely the enzymes responsible for producing more energy in insecticide‐resistant strains and are actively needed to maintain insecticide‐resistance mechanisms [23]. It was previously found that the enzyme increased the resistance of *Culex pipiens pallens* against deltamethrin, both in vitro and in vivo [34]. The differential expression of this protein may thus lead to changes in lipid metabolism in Aba^R^, producing more energy to sustain abamectin resistance mechanisms.

Generally, the proteins described above are related to carbohydrate, lipid, and energy metabolism, and changes in their expression in the Aba^R^ strain have previously been linked to an increase in glycolysis and production of acyl CoA. Insecticide‐resistant strains need more energy to develop resistance mechanisms, such as producing large amounts of detoxifying enzymes to cope with the imposed selection pressure. Lipids are the main source of acetyl CoA for the synthesis of amino acids [23]. Production of more energy to keep these mechanisms active also depends on keeping the Krebs cycle running optimally for better respiration, in addition to keeping the metabolic pathways of various amino acids operational. The Aba^R^ strain seems to show signs of modification and greater adaptability to the imposed pressure introduced by abamectin.

### 4.2 Leloir Pathway Proteins

Galactokinase (Spots 438 and 244, EC 2.7.1.6; functional in Leloir pathway) showed an increased abundance in the Aba^R^ strain. Galactokinase, as a part of carbohydrate metabolism, creates more energy in the Aba^R^ strain to better withstand the selective pressure caused by abamectin [35]. Our finding is consistent with previous studies on permethrin resistance in *C. pipiens pallens* [36] and for phosphine‐treated peach aphids [37].

### 4.3 Alcohol‐Related and Aldehyde Metabolism–Related Proteins

Alcohol dehydrogenase (Spot 280, EC 1.1.1.1), an oxidoreductive enzyme, showed an increased abundance in the Aba^R^ strain compared to the control. The literature presents conflicting data concerning the expression of alcohol dehydrogenase in response to insecticides. For example, *Aedes aegypti* showed up‐regulation [38], whereas *Plodia interpunctella* showed down‐regulation [39].

Aldehyde dehydrogenase (Spot 589, EC 1.2.1.3) catalyzes the conversion of acetaldehyde (as a product of ethanol oxidation) to acetate that can be used in the production of acetyl CoA, a precursor to lipid and amino acid synthesis, and is needed in pesticide‐resistant insect strains. In our study, aldehyde dehydrogenase increased in abundance in Aba^R^, and therefore less acetaldehyde is expected to be produced, leading to greater resistance to abamectin. Aldehyde dehydrogenase is also known to decrease the oxidative stress caused by different compounds, including pesticides.

### 4.4 Sulfur Metabolism–Related Proteins

3(2),5‐Bisphosphate nucleotidase 1 (Spot 32, EC 3.1.3.7) with roles in the synthesis of sulfur amino acids showed decreased in abundance in Aba^R^. Down‐regulation of the corresponding enzyme leads to the production of cysteine. Cysteine is a part of glutathione structure and active site of glutathione‐*S*‐transferase, where a cysteine‐containing tripeptide plays a vital role in cellular antioxidation and detoxification of insecticides [40]. Therefore, a detoxification role and antioxidant defense system can be envisaged for Spot 32 in Aba^R^ strain.

### 4.5 Protein Metabolism–Related Proteins

Cytosol aminopeptidase (Spot 367, EC 3.4.11.14) showed an increase in abundance in response to chemical insecticide pressure. Similar data have been reported for resistant strains in other insects [41, 42]. Our KEGG analysis showed that cytosol aminopeptidase is involved in glutathione and amino acid metabolism. Glutathione is an important part of glutathione‐dependent enzymes, such as glutathione‐*S*‐transferase and glutathione reductase [40], and it may therefore play essential roles in both detoxification and antioxidant defense systems in the Aba^R^ strain.

UV excision repair protein RAD23 homolog B isoform X5 (Spot 414, with a role in the hydrolysis of misfolded proteins) and cathepsin B–like peptidase 2 (Spot 308, EC 3.4.22.1, functioning as a peptide hydrolytic enzyme) showed an increase in abundance in the Aba^R^ strain. Increased abundance of these two enzymes in the Aba^R^ strain may lead to increased synthesis of proteins and enzymes that are involved in resistance to abamectin [43]. Up‐regulation of cathepsin B in resistant strains of *Myzus persicae* and *A. gossypii* to primicarb and spirotetramat has previously been demonstrated [25, 44].

Aminoacylase‐1‐like isoform X1 (Spot 517, EC 3.5.1.14) showed an increase in abundance in Aba^R^ compared to Aba^S^. KEGG analysis showed that aminoacylase plays a significant role in the metabolic processes of ornithine and urea cycles in arginine biosynthesis. Up‐regulation of aminoacylase thus likely leads to the production of more fumarate or arginine. Fumarate is the intermediate compound entering the citrate cycle to synthesize different amino acids to participate in glycolysis, to produce acetyl‐CoA, and eventually more energy. Similarly, exposed *Culex quinquefasciatus* larvae to chlorpyrifos, temephos, and permethrin have been shown to increase arginine concentration [43].

### 4.6 Polyol Pathway Protein for Glucose Metabolism

Aldose reductase (Spot 492, EC 1.1.1.21) reduces toxic aldehydes, formed by reactive oxygen species (ROS), into inactive alcohols that can increase resistance to ROS produced due to toxic effects of abamectin in the Aba^R^ strain. Additionally, increased alcoholic sugars, such as sorbitol, the product of aldose reductase activity, cause innate immune system signaling [45]. This may lead to oxidative stress in the Aba^R^ strain with up‐regulated aldose reductase.

### 4.7 Nucleotide Metabolism–Related Proteins

Adenylosuccinate lyase (Spot 362, EC 4.3.2.2) was increased in abundance in the Aba^R^ strain. This protein is the only enzyme in the purine biosynthetic pathway. Abamectin has been reported to elevate oxidative stresses in insects [46], so we would expect to see such a response in the Aba^R^ strain. Up‐regulation of the enzyme can potentially alleviate DNA damage caused by abamectin in the Aba^R^ strain [47].

### 4.8 Proteins Involved in the Immune System

Among the proteins involved in the immune system, allergen Cr‐PI (Spot 219) and FKBP (Spot 441) were increased in abundance, whereas galectin‐4 (Spot 190), galectin‐9 (Spot 373), and 14‐3‐3‐ zeta (Spot 562) were decreased in abundance in the Aba^R^ strain compared to the Aba^S^ strain.

Allergens belong to a superfamily of lipid‐binding proteins known as lipocalins that participate in cell homeostasis [48]. It has previously been reported to be up‐regulated in different insect strains resistant to pesticides [48, 49]. However, allergen variant 3 was down‐regulated in female salivary glands of pyrethroid‐resistant strains of *A. aegypti* [50]. In response to insecticides, allergens bind to their lipophilic sides, possibly improving the resistance. Allergens can potentially be used as a new target of research on how to overcome insecticide resistance with molecular techniques such as RNAi in WFT.

FKBPs are known as chaperones and co‐chaperones of heat shock proteins that are involved in the regulation of the expression of detoxifying enzymes such as *CYP6B6* in insects [51] and ryanodine receptor Ca^2+^ release channels (RyR), the target site of some new insecticides [52]. Thus, FKBPs would be suitable targets to investigate to overcome resistance to these insecticides.

Galectin‐4, galectin‐9, and 14‐3‐3‐ zeta showed decreased abundance in the Aba^R^ strain compared to the Aba^S^ strain. These proteins are involved in the humoral and cellular immune responses in insects [53]. In the cotton bollworm larvae grown on a diet treated with azadirachtin, down‐regulation of galectins and 14‐3‐3‐ zeta led to reduced immunity [54]. In another experiment, by examining the effects of fipronil and pyraclostrobin on nurse bees, both epsilon and zeta isoforms of 14‐3‐3 were down‐regulated [26]. This suggests that the down‐regulation of galectins and 14‐3‐3‐ zeta in the WFT Aba^R^ strain may lead to a reduced capacity to fight the pathogen. Therefore, the development of insecticide resistance may reduce the insect's subsequent defense potential and provide a negative link between insecticide resistance and susceptibility to insect pathogens in the resistant strain. However, further studies are needed to be carried out to study these ideas in more detail.

### 4.9 Exoskeleton Proteins

Cuticle proteins CPR RR‐1 15 (Spot 193) and larval/pupal cuticle protein H1C (Spot 137) increased in abundance in the Aba^R^ strain compared to the Aba^S^, and CPF 2 (Spot 800) was only expressed in the Aba^R^ strain. Reduced penetration of insecticides through changes in the composition of cuticle and cuticle thickening can improve resistance [55]. Consequently, such mechanisms can delay the arrival of insecticide molecules to the target site and further reduce the bioavailability of insecticides. Increasing the penetration time also improves the functionality of detoxifying enzymes by providing more time to metabolize the insecticides. The up‐regulation of cuticular proteins is mainly associated with endocuticle thickness [44, 56]. The up‐regulation of cuticular proteins in insecticide‐resistant strains of insects has been reported elsewhere [36]. Therefore, it can be concluded that the Aba^R^ strain is likely to have a thicker cuticle than the Aba^S^ strain. This is something that needs to be corroborated with further studies. Our findings indicate that this mechanism may be involved in the resistance to abamectin in the Aba^R^ strain of WFT.

Down‐regulation of ecdysteroid 22‐kinase (Spot 525) leads to the elevation of active ecdysteroids, which is essential in chitin synthesis and cuticle thickening [57]. Similarly, in a lufenuron‐resistant strain of *S. frugiperda*, it was shown that ecdysteroid 22‐kinase is down‐regulated and the resistant strain had higher levels of ecdysteroids as a result of reduced ecdysteroid phosphorylation [58]. Brachyuran (Spot 539, EC 3.4.21.32), a type of serine endopeptidase, hydrolyzes collagen and is a structural protein of the epicuticle component [59]. In our study, brachyuran decreased in abundance in the Aba^R^ strain. Reid [60] obtained similar results and showed that brachyuran experienced a 7.2‐fold down‐regulation in a strain of *C. quinquefasciatus* resistant to permethrin. Down‐regulation of brachyuran leads to reduced collagen metabolism, and, therefore, the amount of collagen in the epicuticle increases, and this may play a role in reducing the cuticle penetration in the Aba^R^ strain.

## 5 Conclusion

The present work is the first attempt to investigate the mechanisms behind abamectin resistance in WFT and quantify DEPs between resistant and susceptible strains of WFT. Our data confirm that changes in proteins (15 DEPs) involved in carbohydrate, lipid, and energy metabolism have major defining roles in abamectin resistance in WFT. Some of the enzymes degrade misfolded proteins to recycle the amino acids in the production of the most needed proteins in Aba^R^ strain to better withstand the pressure caused by abamectin. Furthermore, DNA repair proteins seem to be counter‐defensive in response to abamectin treatment, loading more ROS into the system. Proteins involved in glutathione and arginine biosynthesis seem to play a role in insects resistant to insecticides, and this is a novel finding in our study. Proteins related to the immune system were significantly altered in our study, and that may create a different response to pathogens between resistant and susceptible strains. We describe for the first time the modification of exoskeleton‐related proteins, including cuticular proteins and enzymes involved in the synthesis of the chitin or hydrolysis of the collagen, in association with insecticide resistance in the Aba^R^ strain. We hypothesize that such modifications are in line with the fortification of bodily structures to reduce the penetration of WFT by abamectin as a mechanism of defense in the Aba^R^ strain. Future studies should be focused on the characterization of these proteins and dissect their roles in the toxicity response to abamectin. The present study brought to light many proteins that were not previously thought to be associated with abamectin resistance in *F*. *occidentalis*.

## Author Contributions

**Zahra Gholami**: conceptualization, methodology, formal analysis, investigation, data curation, writing–original draft, writing–review and editing, visualization. **Foad Fatehi**: validation, formal analysis, resources. **Fatemeh Habibpour Mehraban**: formal analysis, investigation, visualization. **Paul A. Haynes**: methodology, resources, writing–review and editing, project administration. **Khalil Talebi Jahromi**: conceptualization, resources. **Vahid Hosseininaveh**: conceptualization. **Hadi Mosallanejad**: conceptualization, resources. **Pär K. Ingvarsson**: resources, writing–review and editing. **Naser Farrokhi**: conceptualization, methodology, validation, formal analysis, resources, writing–review and editing, project administration, supervision.

## Conflicts of Interest

The authors declare no conflicts of interest.

## Data Availability

The data that supports the findings of this study are available in the Supporting Information section of this article.
